# Risk factors of severe pneumonia among children aged 2-59 months in western Kenya: a case control study

**Published:** 2012-11-01

**Authors:** Dickens Onyango, Gideon Kikuvi, Evans Amukoye, Jared Omolo

**Affiliations:** 1 Field Epidemiology and Laboratory Management Training Program, Ministry of Public Health and Sanitation, Kenya; 2 Jomo Kenyatta University of Agriculture and Technology, Kenya; 3 Kenya Medical Research Institute, Director Centre for Respiratory Diseases Research, Nairobi, Kenya

**Keywords:** Risk factors, severe pneumonia, children under five, western Kenya

## Abstract

**Introduction:**

Globally, pneumonia is the leading cause of death in children under the age of 5 years. In Kenya, it is the second leading cause of mortality, accounting for greater than 30,000 deaths in this age group annually. This study sought to identify risk factors for severe pneumonia in children under the age of five years.

**Methods:**

We conducted a case control study. Cases were children aged 2 to59 months with severe pneumonia or very severe pneumonia and controls were those with non-severe pneumonia as defined by the integrated management of childhood illnesses classification. We administered structured questionnaires to mothers of participants to obtain data on socio-demographics, nutritional status and potential environmental risk factors. Data was analyzed using Epi Info; significance level was set at 0.05.

**Results:**

We recruited 103 cases and 103 controls. The median age of cases was 14.0 (Range 3-58) months and of controls 14.0 (Range 2-54) months. Comorbidity (Odds Ratio = 3.8, Confidence Interval 1.4-10.6), delay in seeking treatment for three days or more (Odds Ratio = 2.3, Confidence Interval 1.2-4.2) and contact with upper respiratory tract infection (Odds Ratio = 2.7, Confidence Interval 1.1-6.5) were independent risk factors for severe pneumonia. Receiving antibiotics at home (Odds Ratio = 0.4, Confidence Interval 0.2-0.8) was protective.

**Conclusion:**

Co-morbidity, contact with upper respiratory tract infection and delay in seeking treatment are risk factors for severe pneumonia. We recommend health education regarding appropriate health seeking and engaging community health workers in pneumonia prevention, control and treatment.

## Introduction

Pneumonia is a leading cause of mortality among children under the age of five years globally [1]. The incidence of pneumonia in children under the age of five years is 0.29 episodes per child-year, which equates 151.8 million cases annually in developing countries, a further 4 million cases occur in developed countries. Fifteen countries contribute 74% of the world's annual pneumonia cases [2].

Childhood pneumonia remains a leading killer of children in developing countries where it accounts for up to 21% of deaths in children under the age of five years [3]. The mortality rates of children under the age of five years in most developing countries ranges from 60 to 100 per 1000 live births, one fifth of these deaths are due to pneumonia [4]. An estimated 1.9 million children die from pneumonia yearly [3, 5]. Half the world's deaths due to pneumonia in children under the age of five years occur in Africa [6]. In sub-Saharan Africa, the estimated proportion of death in children aged below 5 years attributed to pneumonia is 17-26% [1]. Kenya is currently ranked among the 15 countries with the highest estimated number of deaths due to clinical pneumonia, the mortality rate being 50.3 per 10, 000 under fives per year [2]. In Kenya, pneumonia is the second leading cause of death among children under the age of five years and causes 16% of deaths in the age group. In 2008 the country had 6,185,800 children under the age of five years, 111,000 of them are estimated to have died, 16% (n = 30,000) of them died of pneumonia [7]. In Kenya, pneumonia in children under the age of five years is currently diagnosed using Integrated Management of Childhood Illness (IMCI) criteria in public health facilities [8].

Three categories of acute respiratory illness based on severity of their clinical presentation are distinguishable by use of physical examination findings. These are: no pneumonia (cough or cold), pneumonia and severe pneumonia [9]. Approximately 13% of pneumonia cases are severe enough to require hospitalization [10]. Of all the pneumonia cases occurring in countries with high incidence, 8.7% are severe enough to be life threatening [2]. Severe pneumonia in childhood is associated with increased long-term respiratory morbidity and disease burden [11] and is more fatal than non severe disease [12]. Understanding the epidemiology of severe pneumonia has been identified as a pressing priority for public health research [13]. Risk factors for pneumonia have been classified into three groups: definite (most evidence consistently pointing to the role of the risk factor); likely (most evidence consistently pointing to the role, but with some opposing findings; or scarce but consistent evidence of the role) and possible (with sporadic and inconsistent reports of the role in some contexts [2]. Studies on the risks factors for severe pneumonia are few in the literature and done more than a decade ago [14, 15].

We conducted this study to identify risk factors for severe pneumonia in children aged 2-59 months presenting with pneumonia in a high volume health facility in Western Kenya.

## Methods

### Study Site

Kakamega Provincial General Hospital is a 449-bed capacity hospital in Western Province, Kenya. It serves as a referral centre for health facilities in the province that has a population of 4,334,282 of whom 2,242,907 are females. About 67.7% of this population lives in rural areas. The province's under five-mortality rate is 121 per 1000 live births, this is the second highest among Kenyan provinces [16].

### Study Design

We conducted a hospital based unmatched case control study. Both cases and controls were recruited at the hospital's casualty department during triage. Case patients were children with severe or very severe pneumonia whereas control patients were children with pneumonia based on the integrated management of childhood illnesses definitions. Severe pneumonia was defined as children less than five years of age with fast breathing and chest wall indrawing. Very severe pneumonia were children with fast breathing and at least one of the following: inability to drink, central cyanosis or any general danger sign, severe respiratory distress or stridor in a calm child. Pneumonia were children with increased respiratory rate and audible crepitations but without features of severe pneumonia. Integrated Management of Childhood Illnesses guidelines were used to define Protein Energy Malnutrition (PEM). Data on the interval between onset of illness and first contact with a health facility was captured by obtaining the respective dates. During bivariate and multivariate analysis, delay was defined as seeking treatment from a health facility after three or more days of onset of illness.

Parents most of whom were mothers of children between 2 and 59 months of age diagnosed with pneumonia were eligible to participate in the study. We collected data on social and demographic factors, nutritional status and environmental risk factors using a structured questionnaire. The Kenya Medical Research Institute (KEMRI) ethical review committee approved the study. We obtained written informed consent from participants after a careful and complete explanation of the study content and purpose.

### Sample Size

The Fleiss formula for calculating sample size for case control studies was used [17]:

n (each group) = (p_0_ q_0_ + p_1_ q_1_)(Z_1-a/2_ + Z_1-b_)^2^/(p_1_- p_0_)^2^

The confidence level was set at 95% and power at 80%. The case to control ratio was 1:1. Using cooking fuel other than liquid petroleum gas was the risk factor used to calculate sample size (Odds Ratio = 2.51, 20.6% of controls are exposed) adapted from Broor et al [15]. This factor is a common occurrence in Kenya, 84% of Kenyans and 97% of the Kenyan rural population use solid fuels for cooking and heating the household [16]. A minimum sample size of 103 cases and 103 controls was obtained. Case patients were selected by systematic random sampling and controls identified sequentially.

### Data Analysis

Data was analyzed using Epi Info version 3.5.3. Bivariate analysis was done followed by unconditional logistic regression for factors with a p value equal to or less than 0.1. Odds ratios and 95% confidence intervals were computed for each exposure variable, the significance level was 0.05.

## Results

One hundred and three case patients and 103 control patients were interviewed. Females made up 50.5% (number = 52) of cases and males were 53.5% (number =54) of controls. Thirty-three percent (number = 34) of case patients and control patients respectively, were aged 3-10 months; the age distribution was comparable in the two study groups. Table 1 shows the socio-demographic characteristics of case and control patients.

**Table 1:** Table 1: socio-demographic characteristics of study participants

Descriptive Variables	Cases		Controls	
	Number (n)	Percent (%)	Number (n)	Percent (%)
**Mother's Marital Status**				
Married	67	65.0%	70	67.9%
Single	28	27.2%	27	26.2%
Divorced	1	1.0%	2	1.9%
Widowed	7	6.8%	4	4.0%
**Mother's Highest Level of Education**				
No formal education	4	3.9%	5	4.9%
Primary	53	51.9%	50	48.6%
Secondary	21	20.6%	28	27.2%
Tertiary	24	23.5%	20	19.4%
**Father's Highest Level of Education**				
No formal education	2	2.7%	6	8.1%
Primary	26	34.6%	26	35.2%
Secondary	20	26.7%	18	24.3%
Tertiary	27	36.0%	24	32.4%
**Mother's Occupation**				
Casual labourer	4	3.9%	3	2.9%
Farmer	17	16.5%	15	14.6%
Housewife	33	32.0%	38	36.9%
Large scale business	3	2.9%	0	0.0%
Salaried employee	20	19.4%	13	12.6%
Small business	19	18.4%	22	21.4%
Student	7	6.8%	12	11.7%
**Father's Occupation**			‘	
Casual labourer	13	17.3%	21	29.2%
Farmer	17	22.7%	13	18.1%
Large scale business	2	2.7%	2	2.8%
Salaried employee	23	30.7%	21	29.2%
Small business	16	21.3%	11	15.3%
Student	4	5.3%	4	5.6%

**Figure 1:**
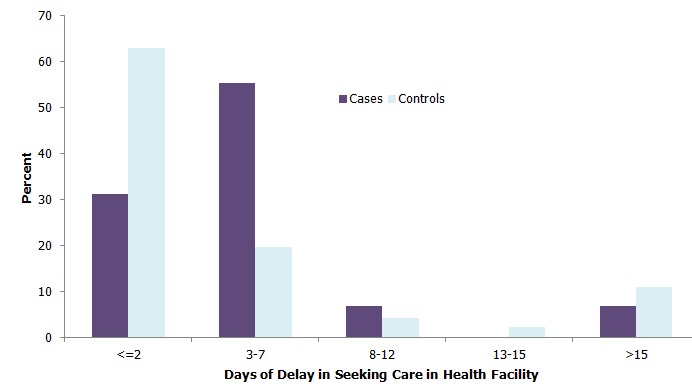
Distribution of study participants by days of delay from onset of illness to seeking treatment

The median delay from onset of illness to the time of seeking treatment in a health facility was 4 days (interquartile range = 4) for cases and 2 days (interquartile range = 5) for controls. Thirty-one percent (number = 32) of cases sought treatment within two days, 55.3% (number = 57) took 3 to 7 days and 6.8% (number = 7) took more than 15 days. Sixty-three percent (number = 58) of controls sought treatment within 2 days and 19.6% (number = 18) within 3 to 7 days. Figure 1 shows the distribution of cases and controls by number of days of delay in seeking medical treatment.

Sixty-five percent (number = 67) of cases and 53.6% (number = 52) of controls reported having taken medication at home. Of the cases who had received medication at home, 22.4% (number = 15) had taken antibiotics compared to 62% (number = 32) of controls. Eighteen percent (number = 19) of cases and 7% (number = 7) of controls had comorbidities. Eight percent (number = 8) of cases had protein energy malnutrition and another 8% (number = 8) had human immunodeficiency virus infection. Six percent (number = 6) of controls had human immunodeficiency virus infection and 1% (number = 1) had protein energy malnutrition.

Forty-five percent (number = 44) of cases were aware of community health workers in their neighbourhood compared to 48.0% (number = 47) of controls. Twenty-eight percent (number = 31) of cases reported having received assistance from a community health worker during the current illness compared to 26% (number = 27) of controls.

On bivariate analysis, socio-demographic factors were not significant. Those who sought herbal treatment before visiting a health facility (Odds Ratio = 3.41, Confidence Interval-1.45-8.05), and those who had a comorbidity (Odds Ratio = 3.10, Confidence Interval-1.24-7.74) were more likely to have severe pneumonia. Similarly, those who had contact with a relative suffering from upper respiratory tract infection (Odds Ratio =2.82, Confidence Interval -1.27-6.26), whose nearest health facility was more than five kilometers away (OR = 1.80, CI-1.01-3.19) and those who delayed to seek medical treatment for 3 days or more (Odds Ratio = 2.86, Confidence Interval -1.62-5.06) were more likely to have severe pneumonia. Those who had received antibiotics at home (Odds Ratio = 0.38, Confidence Interval - 0.19-0.75) were less likely to have severe pneumonia. Table 2 shows results of bivariate analysis.

**Table 2:** Bivariate analysis of risk factors for severe pneumonia in a group of children aged 2-59 months in western Kenya

Variables	Cases Number (Column Percent%)	Controls Number (Column Percent%)	Odds Ratio (95% Confidence Interval)	*P* value
**Exclusive Breastfeeding for 6 mont**hs				
Yes	58 (56.3)	64 (64.0)	0.73 (0.41-1.27)	0.33
No	45 (43.7)	36 (36.0)	Ref	
**Initial Action on the Illne**ss				
Visited herbalist	23 (22.3)	8 (7.8)	3.41 (1.45-8.05)	0.01
Visited health facility/pharmacy	80 (77.7)	95 (92.2)	Ref	
**Hospitalized with Diarrhea in the Last 6 Months**				
Yes	23 (22.3)	12 (11.7)	2.18 (1.01-4.66)	0.06
No	80 (77.7)	91 (88.3)	Ref	
**Has Comorbidity**				
Yes	19 (18.4)	7 (6.8)	3.10 (1.24-7.74)	0.02
No	84 (81.6)	96 (93.2)	Ref	
**Has Malnutrition**				
Yes	8 (7.8)	1 (1.0)	8.59 (1.05-69.9)	0.04
No	95 (92.2)	102 (99.0)	Ref	
**Has Human Immunodeficiency Virus Infection**				
Yes	8 (7.8)	6 (5.8)	1.36 (0.46-4.07)	0.78
No	95 (92.2)	97 (94.2)	Ref	
**Contact with Member of Household with upper respiratory tract infection**				
Yes	24 (23.3)	10 (9.7)	2.82 (1.27-6.26)	0.01
No	79 (76.7)	93 (90.3)	Ref	
**Vaccination Status Appropriate for Age**				
Yes	75 (73.5)	76 (76.0)	0.88 (0.46-1.66)	0.81
No	27 (26.5)	24 (24.0)	Ref	
**Household Fue**l				
Liquefied Petroleum Gas	17 (16.5)	27 (26.2)	0.56 (0.28-1.09)	0.13
Non Liquefied Petroleum Gas	86 (83.5)	76 (73.8)	Ref	
**Delayed 3 or more days in Seeking Care from Health Facility**				
Yes	71 (68.9)	45 (43.7)	2.86 (1.62-5.06)	0.01
No	32 (31.1)	58 (56.3)		
**Nearest Health Facility More Than 5 kilometres away**				
Yes	72 (69.9)	58 (56.3)	1.80 (1.01-3.19)	0.06
No	31 (30.1)	45 (43.7)	Ref	
**Received Antibiotic at Home**				
Yes	15 (14.6)	32 (31.1)	0.38 (0.19-0.75)	0.01
No	88 (85.4)	71 (68.9)	Ref	
**Refusal to Feed as Part of Symptoms**				
Yes	67 (65.0)	51 (49.5)	1.90 (1.08-3.32)	0.03
No	36 (35.0)	52 (50.5)	Ref	

The following were independent risk factors for severe pneumonia after controlling for multiple potential confounders: comorbidity (Odds Ratio = 3.8, Confidence Interval -1.4-10.6), delay in seeking medical treatment for three days or more (Odds Ratio =2.3, Confidence Interval -1.2-4.2) and contact with a household member with upper respiratory tract infection (Odds Ratio = 2.7, Confidence Interval-1.1-6.5). Receiving antibiotics at home (Odds Ratio = 0.4, Confidence Interval-0.2-0.8) was protective. Table 3 shows results of multivariate analysis.

**Table 3:** Unconditional Logistic Regression of Risk Factors of Severe Pneumonia

Risk Factors	Adjusted Odds Ratio	95% Confidence Interval	P-Value
Having a comorbidity	3.79	1.36-10.58	0.01
Delay for 3 or more days to seek Treatment in health facility	2.26	1.23-4.18	<0.01
Contact with member of the household with upper respiratory tract infection	2.71	1.12-6.52	0.03
Received antibiotics at home	0.39	0.19-0.85	0.02

## Discussion

This study investigated risk factors for children having severe pneumonia or very severe pneumonia at their first presentation in a high volume public hospital's casualty. Independent risk factors for severe pneumonia were contact with a household member with upper respiratory tract infection symptoms, comorbidity and delay in seeking medical treatment in a health facility. Having taken antibiotics prior to the hospital visit was protective.

Those who had a member of their household with upper respiratory symptoms were about three times more likely to have severe pneumonia. A study by Broor et al had similar findings, disease in the mother had an odds ratio of 6.53 whereas if the diseased person was a sibling the odds ratio was 24 [15]. Upper respiratory tract infections are very contagious and are easily transmitted from household contacts to children. These infections are often viral in origin and predispose children to pneumonia [18]. Severity of the disease also depends on virulence and load of the pathogen; the load is usually higher when infection is from a household contact [19].

Children who delayed to seek medical treatment in a health facility by three days or more were twice more likely to present with severe pneumonia. This finding is consistent with a study in Uganda that found the median duration of illness before care is sought to be seven days [20]. Another study in Uganda also found the long delay in seeking medical care with majority of the respondents having tried home medication before visiting the health facility. This delay was in spite of the fact that there was prompt recognition of symptoms [21]. The progress of pneumonia is rapid and delayed intervention can lead to increased disease severity and even death [22]. The possible explanation for this delay is the various pre-hospital home treatments that children get before seeking care in a health facility. The treatments given in these circumstances are often inappropriate or inadequate. These findings are consistent with a WHO report which has identified the delay in seeking care as children are treated at home through the informal sector or by traditional healers as an important barrier to the reduction of mortality in children [23].

Those with comorbidity were four times more likely to have severe pneumonia. Children who have a concomitant chronic illness may have their immunity lowered making them more susceptible to severe disease. Suwanjutha et al found that children with an underlying heart condition were four times more likely to have severe pneumonia [24]. Concomitant presence of protein energy malnutrition has been reported as a risk factor by some studies [15, 25] whereas others have reported lack of association [24]. In cases where the comorbidity happens to be human immunodeficiency virus, studies have shown that children who are human immunodeficiency virus infected are 40 times more likely to get pneumonia than their HIV free counterparts are. Furthermore, they are prone to infection by atypical organisms and antibiotic resistant strains [26, 27]. This study combined all the concomitant diseases as opposed to the above that considered the individual role of the concomitant diseases but our finding is in consonance with these earlier results. Rudan et al in their metaanalysis lists the presence of concomitant diseases as one of the likely risk factors for pneumonia; most evidence consistently point to the role, but there are some opposing findings [2]. Our finding, therefore, adds to the body of evidence that point to the role of comorbidity as a risk factor for the severity of pneumonia.

Home medication was quite common in this study. Receiving antibiotics at home reduced the risk of severe pneumonia. This result is comparable to a study in Uganda where 23% had received antibiotics alone and another 25% had received antibiotics in combination with antimalarials. Those who received antimalarials only were 5.5 times more likely to have severe pneumonia [21]. This finding by Hildenwall et al implies that receiving antibiotics at home protects children from severe pneumonia. In 2004, World Health Organisation recommended the treatment of non-severe pneumonia with oral antibiotics by trained community health workers at the community level [23]. A metaanalysis of clinical trials on community case management reported a 36% decline in child mortality from pneumonia when trained community health workers administered antibiotics to children under the age of five years with pneumonia [28]. An update to the above metaanalysis estimates that community case management can reduce pneumonia mortality by about 70% in children under the age of five years [29]. A recent randomized clinical trial done in rural Pakistan in which children with severe pneumonia were randomized to receive oral amoxicillin at home or cotrimoxazole and referral to a health facility showed similar levels of treatment failure in the two groups [30]. This finding suggests that severe pneumonia can be successfully treated at the community level with oral amoxicillin in resource poor settings. In spite of this overwhelming body of evidence, many developing countries including Kenya have no policy to permit and implement community case management. Kenya is estimated to have a pneumonia treatment gap of 804,000 per year in children aged under five years [31] which can be reduced by community case management implementation. A trial or pilot of CCM in our set up maybe necessary to find out if the results can be replicated locally.

## Conclusion

Co-morbidity, contact with upper respiratory tract infection and delay in seeking appropriate treatment are the main risk factors for severe pneumonia while receiving antibiotics at home is protective. We recommend more health education regarding appropriate health seeking and greater interventions at the community level by engaging community health workers in pneumonia prevention, control and treatment.
